# Functional Characterization of *khadi* Yeasts Isolates for Selection of Starter Cultures

**DOI:** 10.4014/jmb.2109.09003

**Published:** 2021-12-01

**Authors:** Koketso Motlhanka, Kebaneilwe Lebani, Mar Garcia-Aloy, Nerve Zhou

**Affiliations:** 1 Department of Biological Sciences and Biotechnology, Botswana International University of Science and Technology, Private Bag 16, Central District, Palapye, Botswana; 2 Metabolomics Unit, Food Quality and Nutrition Department, Research and Innovation Centre, Fondazione Edmund Mach, via E. Mach 1, 38098 San Michele all’Adige, Italy

**Keywords:** *Khadi*, microbial characterization, fermentation profiling, aroma profiling

## Abstract

Yeasts play an important role in spontaneous fermentation of traditional alcoholic beverages. Our previous study revealed that a mixed-consortia of both *Saccharomyces* and non-*Saccharomyces* yeasts were responsible for fermentation of *khadi*, a popular, non-standardized traditional beverage with an immense potential for commercialization in Botswana. Functional characterization of isolated fermenting yeasts from mixed consortia is an indispensable step towards the selection of potential starter cultures for commercialization of *khadi*. In this study, we report the characterization of 13 *khadi* isolates for the presence of brewing-relevant phenotypes such as their fermentative capacity, ability to utilize a range of carbon sources and their ability to withstand brewing-associated stresses, as a principal step towards selection of starter cultures. *Khadi* isolates such as *Saccharomyces cerevisiae*, *Saccharomycodes ludwigii* and *Candida ethanolica* showed good brewing credentials but *Lachancea fermentati* emerged as the isolate with the best brewing attributes with a potential as a starter culture. However, we were then prompted to investigate the potential of *L. fermentati* to influence the fruity aromatic flavor, characteristic of *khadi*. The aroma components of 18 *khadi* samples were extracted using headspace solid phase micro-extraction (HSSPME) and identified using a GC-MS. We detected esters as the majority of volatile compounds in *khadi*, typical of the aromatic signature of both *khadi* and *L. fermentati* associated fermentations. This work shows that *L. fermentati* has potential for commercial production of *khadi*.

## Introduction

*Khadi* is a traditional alcoholic beverage produced in Botswana for household consumption and as a source of subsistence. The distinctive aroma complexity and popularity of this traditional beverage among the people of Botswana has increased interest in its commercialization. However, there has been little documentation and information on the production processes to brew *khadi*. Previous work done by Motlhanka, Zhou and Lebani in 2018 [1] is the first work to document the brewing of *khadi*, described it as a fermentation product of ripened and sun-dried *Grewia flava* (*Malvaceae*) fruits supplemented with brown table sugar. Brewers employ a back-slopping technique in which the previously fermented fruits are pitched as starter cultures for the next fermentation step. Uncontrolled spontaneous fermentation, unstandardized brewing processes, unsafe practices such as the use of marijuana, car battery acid, tobacco, and even dagga are documented as responsible for product inconsistencies and food poisoning [2, 3]. A subsequent study by Motlhanka *et al*. (2020) documented the dominance of both *Saccharomyces* and non-*Saccharomyces* yeasts in *khadi*, suggesting their important role in the production of this beverage. Functional characterization of *khadi* yeast isolates is one of the crucial lead steps towards the development of starter cultures.

Starter cultures are crucial in current brewing trends towards standardized processes to produce high quality and safe beverages if the product is to make its way into the modern markets. Highly productive yeast strains with a wider substrate utilization base, brewing-associated stress tolerance as well as production of distinct aromas are an ideal requirement for commercial production strains. In addition, consumers in the modern world whose advanced lifestyles are changing, thereby leading to demand for selection or development of strains with novel traits [4, 5]. As such, traditional alcoholic beverages are a potential source of new fermentative microorganisms whose fermentation profile is responsible for the distinctive aroma complexities.

There is little information on aroma profiles [2] and no information on potential starter cultures of *khadi* published to date. Aromatic profiling of *khadi* and subsequent functional characterization of fermenting yeast isolates are important steps towards selection and development of starter cultures for a fully standardized production process. This study describes the aroma profiles of 18 *khadi* samples reported in our previous work [6]. In addition, we characterized the functional traits of 13 fermentative yeasts (isolated from *khadi* as reported [6]) for their utility as starter cultures for the production of a commercial grade *khadi*. We assessed their ability to assimilate and ferment different sugars used for industrial fermentations. As the ability to tolerate brewing-associated stresses is an important trait for efficient industrial fermentations, we further assessed their osmotolerance, thermo-tolerance, tolerance to varying concentrations of acids and ethanol compared to selected industrial *Saccharomyces* yeasts. The results were then used to select for yeast with the most potential as a starter culture for *khadi* brewing.

## Materials and Methods

### *Khadi* Samples and Yeast Isolates

*Khadi* samples were collected from 18 local *khadi* brewers from a variety of locations in Botswana (Tonota, Palapye, Serowe, Letlhakane, Mmashoro and Maun) as previously described [6]. A total of 13 yeast isolates previously isolated from the 18 *khadi* samples (Table 1) [6] were selected for assessment as potential starter cultures. Fermentative traits of the isolates were compared to four commercial brewing yeasts — *S. cerevisiae* (SafAle T-58, Fermentis, France), lager yeast — *S. pastorianus*, (Lallemand Brewing, Austria), wine yeast — *S. cerevisiae*, (Lalvin EC-1118, Lallemand Brewing) and baker’s yeast — *S. cerevisiae* (Anchor yeast, South Africa) as controls.

### Functional Characterization

**Carbon assimilation assay.** Assimilation of a wide range of substrates, an important attribute of starter cultures enabling attenuation and economical production, was tested using an API 20C AUX kit (Bio-Mérieux, Marcy l'Etoile, France) according to the manufacturer's instructions. In addition, the yeast isolates were also tested for their ability to utilize industrial carbon sources (glucose, maltose, sucrose, fructose and lactose) to further characterize their growth credentials on these sugars. In brief, as ingle colony was picked and used to prepare seed cultures by inoculating into 2 ml of YPD broth in 5 ml tubes and grown overnight in a shaking incubator at 30°C and 180 rpm. The cells were then harvested by spinning down at 4,000 rpm for 2 min and washed with sterile deionized water. The cells were then adjusted to an OD_600nm_ of 0.5 using phosphate buffered saline 1%. The cultures were further diluted in 96-well plates to give working 2-fold dilutions (OD_600nm_ of 0.5, 0.25 and 0.125). Cells in these dilution ranges were then spotted using an 8*6 array stainless steel replicator (spot stamp test) on different solid media plates containing Yeast extract (1%) and Peptone (2%) supplemented with different sugars [maltose (2%), fructose (2%), sucrose (10% and 20%), glucose (2%) and lactose (2%)]. The plates were incubated at 30°C, photographed and recorded every day. Ability to assimilate respective sugars was determined semi-quantitatively by scoring the growth on a control plate (with YPD) in comparison to those that grew on a plate with a tested sugar. The spot test was repeated twice in triplicates, starting from independent cultures.

**Determination of fermentative capacity.** Ability to ferment reducing sugars into ethanol, as an indispensable attribute of a yeast starter cultures in brewing was tested as reported by [7] with minor adjustments. In brief, a single colony from potential strains was grown overnight in media containing *G. flava* fruits (200 g/l) and brown sugar (100 g/l) (GFS media) in 5 ml test tubes. The samples were incubated at 30°C with a shaking speed of 180 rpm. Overnight cultures were pelleted, washed with sterile deionized water and used for inoculation in GFS media. The GFS media (3 ml) was inoculated with a yeast suspension culture to give an initial OD_600nm_ of 1.0 and introduced into a 30 ml marked syringes in triplicates. The syringes were incubated in a shaker at 30°C at 180 rpm for 24 h. Fermentative capacity was determined by measuring carbon dioxide evolved during fermentation, which corresponded to the movement of the syringe plunger. The results were recorded at hourly intervals until fermentation was complete, that is, after the plunger fell off the syringes. The ale yeast, baker’s yeast and wine yeast were included as controls. The experiments were repeated twice in triplicates.

**Determination of brewing associated-stress tolerance.** Brewing-associated stress tolerance, another important attribute for starter cultures, known to impact their fermentative performance and subsequently the overall quality of a beverage was investigated by exposing yeasts to osmotic, ethanol, pH, and high temperature fermentation stresses. Potential starter culture isolates from *G. flava* fruits and *khadi* used were inoculated in YPD broth in 5 ml tubes and grown overnight in a shaking incubator at 30°C and 180 ×*g*. The cells were harvested by spinning down at 13,000 ×*g* for 2 min and washed with deionized sterile water. The OD_600nm_ of cells corresponding to each isolate were adjusted, diluted and spotted as described in Section 2.21 except that solid media plates containing YPD supplemented with different stressors at different concentrations to test for stress tolerance were used. For osmotolerance, cells were spotted on YPD with different NaCl concentrations (0.5 M, 1 M, 1.5 M, 2 M) and a control without NaCl. The plates were incubated at 30°C and photographed and recorded every day. Stress tolerance was determined semi-quantitatively by scoring the growth on a control plate (without NaCl) in comparison to those that grew on a plate with NaCl. Ethanol stress tolerance was tested as above except those cells were spotted on YPD plates containing different ethanol concentrations (3, 5, 7, 9 and a control with 0% ethanol). pH stress tolerance was also tested as above except those yeasts were grown in liquid media sets at different pH (1, 2, 3, 5 and a control pH of 6.2) and then spotted on YPD plates. To test for thermo-tolerance, isolates were spotted on YPD plates and incubated at different temperatures (37, 40, 41, 42, 43 and a control temperature of 30°C) and growth was scored as above. All spot tests were repeated twice in triplicates, starting from independent cultures.

### Aroma Profiling

**HS-SPME method.** Headspace solid phase micro-extraction (HS-SPME) is a solvent free method that incorporates three steps namely; sampling, extraction and concentration of analytes. Eight ml of sample was measured into a sampling vial then heated to about 60°C in a water-bath for approximately 30 min. The SPME Divinylbenzene/Carboxen/Polydimethylsiloxane (DVD/CAR/PDMS) fiber was introduced into the sample headspace and allowed to stand for approximately 30 min in order to sample, extract and concentrate the volatiles. The SPME fiber was manually introduced into GC injector for about 5 min to allow desorption of analytes.

**GC-MS analysis.** Chromatographic analyses were performed using an Agilent 6890 N gas chromatograph equipped with a 5973 N mass spectrometer (Agilent, USA). The target analytes were separated using a TG-WAXMS column (30 m × 0.25 mm × 0.25 μm film thickness) (Agilent) with helium as a gas carrier at 34 cm/s average linear velocity with a constant carrier flow of 1.5ml/min. The column was maintained at 40°C for 5 min after desorption, ramped at 4°C/min up to 200°C, and then ramped at 10°C/min up to 240°C, where it was held for 15 min. The oven temperature was programmed as follows: 1 min at 35°C then rising to 240°C at a rate of 10°C per min, with a total run time of 24 min. All mass spectra were acquired in electron impact (EI) mode at 70 eV, using full scan with a scan range of 30 – 400 atomic mass units, at a rate of 6.12 scans/s. Identification of compounds was obtained by comparing the retention times with those of authentic compounds and the spectral data from NIST libraries (https://www.nist.gov/nist-research-library). The work on section 2.1.2 and 2.1.3 was performed at Botswana Institute for Technology Research and Innovation (BITRI).

### Statistical and Cluster Analyses

The differences in the gassing power between each *khadi* isolate and the control bakers’ yeast, wine yeast, ale yeast and lager yeast were analyzed using STATISTICA, version 13.2 (Statsoft Inc., Japan). The variables were compared using a one-way ANOVA and a post-hoc Tukey HSD (*p* < 0.05) test for multiple comparisons. The clustering of aroma compounds, carbon assimilation results and stress tolerance results were performed using the R package "pheatmap". For quantitative data, values were scaled to prevent the output being dominated by the largest values.

## Results and Discussion

### Functional Characterization of *khadi* Isolates Reveal Their Potential as Starter Cultures

As a crucial step towards the development of starter cultures for reproducible bioprocessing, functional tests such as their ability to assimilate a wide variety of carbon sources, ability to ferment those carbon sources as well as ability to tolerate brewing associated stress *i.e.*, osmotic stress, thermal stress, oxidative stress and fluctuations in acidity or basicity (pH) stress were carried out. These tests were done because there are several stresses that microbes encounter during fermentation thus, it is pertinent to select the best performing strain to be used for the bioprocess development of *khadi*.

**Fig. 1:**
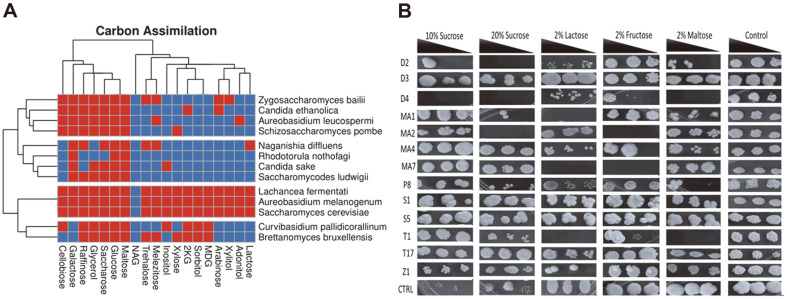
Carbon assimilation of the yeast isolates from *G. flava* fruits and *khadi* samples. (**A**) The carbon assimilation of the yeast different yeasts on an API 20 kit. Red shows that the yeast isolate could utilize the carbon source while blue is for isolates that could not utilize the carbon source in the API kit. Key: 2KG = Calcium 2-keto-Gluconate, MDG = Methyl-α-Glucopyranoside and NAG = N-Acetyl-Glucosamine. (**B**) Ability of the isolated yeast strains to utilize different carbon sources. The baker’s yeast (*S. cerevisiae*) was used at the control (CTRL) since it is one of the strains that *khadi* brewers use. The black triangle shows concentrations of the dilutions from left to right (0.5, 0.25 and 0.125). Key: D2 (*Aureobasidium leucospermi*), D3 (*Aureobasidium melanogenum*), D4 (*Naganishia diffluens*), MA1 (*Saccharomyces cerevisiae*), MA2 (*Candida sake*), MA4 (*Lachancea fermentati*), MA7 (*Curvibasidium pallidicorallinum*), P8 (*Schizosaccharomyces pombe*), S1 (*Saccharomycodes ludwigii*), S5 (*Brettanomyces bruxellensis*), T1 (*Zygosaccharomyces bailii*), T17 (*Candida ethanolica*) and Z1 (*Rhodotorula nothofagi*). Plates were incubated 3 days at 30°C in triplicates and representative plates were photographed and reported.

*Khadi* isolates exhibit a wide carbon substrate assimilation range. Carbon substrate assimilation assays revealed the isolates were capable of assimilating a wide range of carbon sources (Fig. 1). Although the utilization of wide substrate range is one of the important attributes for potential brewing starter cultures, assimilation of sucrose is the most important in *khadi* brewing. Sucrose (added as brown sugar) is the main sugar available to yeasts during *khadi* brewing. All isolates assimilate sucrose except *Rhodotorula nothofagi*. In this regard all isolates have potential as starter cultures for *khadi* brewing. It is noteworthy that *Grewia* fruits contain 28–30% (w/w) reducing sugars and 27–29% (w/w) sucrose (Sati and Ahmed, 2018). The heatmap (Fig. 1) is divided into 4 clusters where the *Lachancea fermentati* cluster is for the isolates that could utilize all the carbon sources except NAG. Our results further show that most of these isolates could assimilate reducing sugars such as D-glucose, D-galactose, D-maltose, D-lactose, D-xylose, and D-cellobiose and thus yeasts capable of converting these sugars into ethanol will be ideal as starter cultures. Ability to ferment carbon sources is important for fermentation productivity and attenuation where both are responsible for the level of alcohol production.

The above results from the carbon assimilation through the use of an API kit prompted us to further characterize the extent of assimilation of carbon sources found in *Grewia* spp. fruits. In addition, we also sought to investigate the possibility of using alternative carbon sources as alternatives to sucrose used for brewing of *khadi*. The isolates were grown on different carbon sources namely maltose, fructose, lactose and sucrose at different concentrations as a control. Most of the isolates grew well at 10% (v/v) sucrose. However, we noted that *N. diffluens*, a yeast we isolated from *G. flava* fruits could not utilize sucrose. The result is not that surprising, as this yeast is a homonym of *Cryptococcus diffluens* [8], a human finger nail yeast whose ecological niche is not fruits. The control baker’s yeast strain had an average growth at 20% sucrose (Fig. 1B) which is to be expected as the strain is used in high sugar concentration doughs with an average sucrose level of approximately 30% [9].

Isolates *A. melanogenum* and control baker’s yeast strain grew very well on the lactose media while *A. leucospermi*, *N. diffluens*, *L. fermentati* and *R. nothofagi* showed poor growth. Lactose is a disaccharide, made up of galactose and glucose, whose utilization depends on the presence of the *LAC4* genes which can break lactose into its monomers and research has shown that most yeasts reported in alcoholic milk fermented products lack genes encoding β-*galactosidases*, suggesting that they must rely on free monosaccharides or lactic acid [10]. Fructose (fruit sugar) assimilation is a desirable trait as it is abundant in fruits. Our results show that all the strains utilized fructose except for MA2 (*C. sake*) and MA7 (*C. pallidicorallinum*). In addition, utilization of maltose is a preferable trait if biomass production will be carried out using molasses, as it is the case with industrial baker’s yeast production. The isolates D4 (*N. diffluens*) and T1 (*Z. bailii*) were not able to grow on media with maltose while the control strain; D3 (*A. melanogenum*), T17 (*C. ethanolica*) and S5 (*B. bruxellensis*) grew very well.

**Fig. 2:**
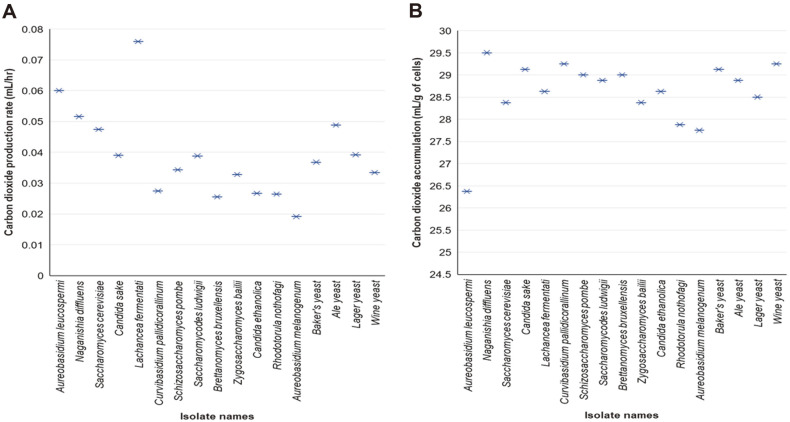
Carbon dioxide production rate of yeast isolates. (**A**) The average carbon dioxide production rates (ml/h) of the yeast isolates and the industrial yeasts (ale, baker’s, lager and wine yeasts) were plotted. The CO_2_ production rate ranged from 0.019 ± 0.001 to 0.076 ± 0.004ml/h while the CO_2_ yield ranged from 27.8 ± 2.1 to 29.5 ± 0.4 ml/ g of cells. The Tukey’s HSD test results (isolated yeasts vs baker’s yeast) are presented with asterisks (*p < 0.05), **p < 0.01 and ***p < 0.001). (**B**) Total amount of CO_2_ accumulated at the end of fermentation. There were no statistical differences in the accumulation of CO_2_ except for *A. leucospermi* when compared the baker’s yeast. CO_2_ yield correlates to the amount of ethanol produced [11, 12].

**Elevated fermentation capacity of *khadi* isolates: an indispensable attribute of potential starter cultures.** Alcoholic fermentation is an indispensable attribute for selection of potential starter cultures. Determination of fermentative capacity (*i.e.*, rate of CO_2_ production during fermentation) [11, 12] was done using syringes and synthetic media prepared using *G. flava* fruits as a source of nutrients and brown sugar as a carbon source mimicking the *khadi* brewing process. The results show that *A. leucospermi*, *A. melanogenum*, *S. cerevisiae* and *L. fermentati* exhibited a significantly higher CO_2_ production rate when compared to the commercial baker’s yeast (Tukey’s HSD test; *p* < 0.001) (Table SA2). *L. fermentati* (MA4) had a CO_2_ production rate of 0.076 ± 0.004 ml/h which was significantly higher than all the isolates and the four industrial yeasts controls (Fig. 2A)(Supplementary Materials). *L. fermentati* (MA4) is the most ideal strain to use for the fermentation of *khadi* based on the fermentative capacity as an attribute for selection of the most desirable starter culture. Taking into account that the total amount of CO_2_ accumulated at the end of fermentation (CO_2_ yield) correlates to the amount of ethanol produced (every mole of CO_2_ produced, there is 1 M of ethanol produced) [11, 12], our results suggest that most isolates are ideal as starter cultures except *A. leucospermi* (Fig. 2A, Table SA4). The final concentration of ethanol of the final product almost the same as the ethanol concentration used in the ethanol tolerance experiment.

**Fig. 3:**
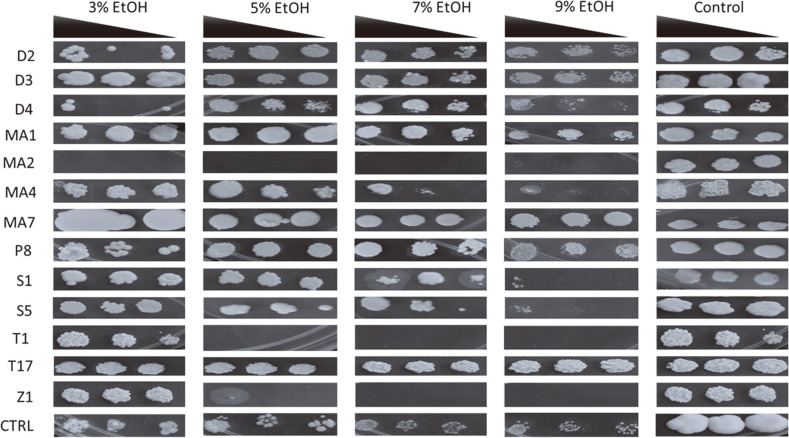
Growth inhibition of the yeast strains due to different ethanol concentrations. The baker’s yeast (*S. cerevisiae*) was used as the control (CTRL) since it is a strain commonly found in *khadi*. The black triangle shows concentrations of the dilutions from left to right (0.5, 0.25 and 0.125). Key: D2 (*Aureobasidium leucospermi*), D3 (*Aureobasidium melanogenum*), D4 (*Naganishia diffluens*), MA1 (*Saccharomyces cerevisiae*), MA2 (*Candida sake*), MA4 (*Lachancea fermentati*), MA7 (*Curvibasidium pallidicorallinum*), P8 (*Schizosaccharomyces pombe*), S1 (*Saccharomycodes ludwigii*), S5 (*Brettanomyces bruxellensis*), T1 (*Zygosaccharomyces bailii*), T17 (*Candida ethanolica*) and Z1 (*Rhodotorula nothofagi*). Plates were incubated 3 days at 30°C in triplicates and representative plates were photographed and reported.

**Ethanol stress tolerance of *khadi* isolates reveal their robustness as starter cultures.** As ethanol is accumulated during alcoholic fermentation, the rate of its production decreases progressively due to dose dependent inhibition of cellular processes. High concentrations of ethanol (strain specific), denature glycolytic enzymes [13, 14], and deface cell membranes leading to disruption of their functions such as transportation of sugars required for ethanol production. In addition, ethanol overall reduces growth of yeast cells, reduces cell vitality and increases cell death (Birch and Walker 2000). As such, the fermentation process requires a strain that can withstand elevated ethanol concentrations without its growth being inhibited or impaired. All potential starter culture isolates except MA2 (*C. sake*) were not affected by the presence of 3% ethanol as their growth was comparable to when grown in the absence of ethanol (Fig. 3). It is noteworthy that ethanol concentration of *khadi* ranges from about 3.69% (*v/v*) (Mapitse *et al*., 2014) to an average of 5.27 ± 2.02% [6]. All isolates were tolerant to 5% ethanol except Z1 (*R. nothofagi*) and MA2 (*C. sake*) as seen in the heatmap. Ethanol tolerance was also tested at 9% ethanol, because our previous work recorded an outlier with about 9% ethanol concentrations in [6]. All the isolates, except MA2 (*C. sake*), T1 (*Z. bailii*), and Z1 (*R. nothofagi*), remained tolerant to 9% ethanol suggesting that all these isolates (with the three listed exceptions) have potential to be used as starter cultures to produce higher amounts of ethanol without a decrease in productivity.

**Fig. 4:**
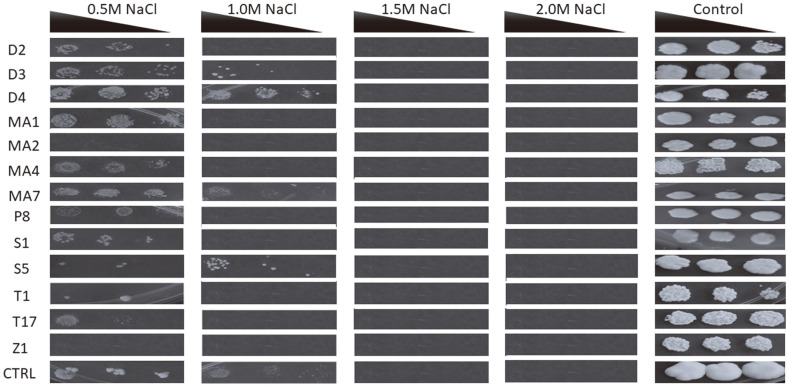
Osmo-tolerance of the yeast isolates due to supplementation with various NaCl concentrations. The baker’s yeast (*S. cerevisiae*) was used as the control (CTRL) since it is a strain commonly found in *khadi*. The black triangle shows concentrations of the dilutions from left to right (OD_600nm_ 0.5, 0.25 and 0.125). Key: D2 (*Aureobasidium leucospermi*), D3 (*Aureobasidium melanogenum*), D4 (*Naganishia diffluens*), MA1 (*Saccharomyces cerevisiae*), MA2 (*Candida sake*), MA4 (*Lachancea fermentati*), MA7 (*Curvibasidium pallidicorallinum*), P8 (*Schizosaccharomyces pombe*), S1 (*Saccharomycodes ludwigii*), S5 (*Brettanomyces bruxellensis*), T1 (*Zygosaccharomyces bailii*), T17 (*Candida ethanolica*) and Z1 (*Rhodotorula nothofagi*). Plates were incubated 3 days at 30°C in triplicates and representative plates were photographed and reported.

**Osmotolerance is not well pronounced among *khadi* isolates.** The ability of a starter culture to tolerate increased amounts of sugar in the substrate, which is associated with a higher osmotic stress, is an added advantage. The amounts of fruit sugars fluctuates from season to season [15] and during processing [16]. Since osmotic stress reduces productivity of production strains, utilization of osmotolerant strains is an important attribute in potential brewing starter cultures. Our results suggest that all isolates tolerated the presence of an osmolyte (0.5M NaCl) except for MA2 (*C. sake*) and Z1 (*R. nothofagi*) (Fig. 4). However, increasing the osmotic pressure by growing isolates on 1.0 M NaCl was inhibitory for growth of most isolates except D3 (*A. melanogenum*), D4 (*N. diffluens*), MA4 (*L. fermentati*), S1 (*S. ludwigii*) and the control baker’s yeast. These results suggest that strains will be highly impaired or inhibited by media with high sugar concentrations.

**Fig. 5:**
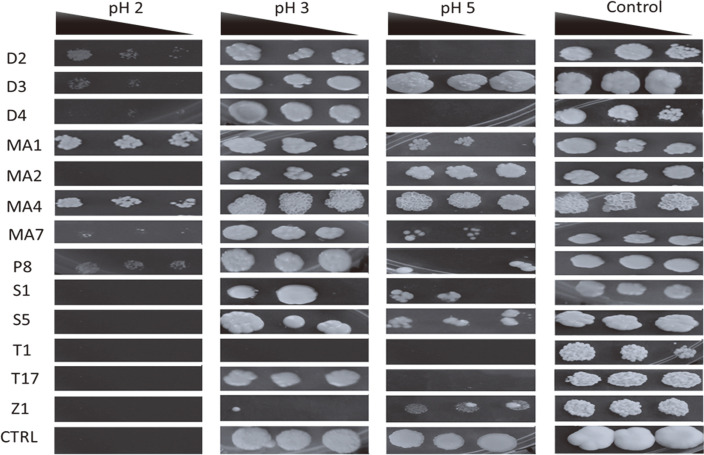
pH stress tolerance of yeast isolates. The baker’s yeast (*S. cerevisiae*) was used as the control (CTRL) since it is a strain commonly found in *khadi*. The black triangle shows concentrations of the dilutions from left to right (OD_600nm_ 0.5, 0.25 and 0.125). Key: D2 (*Aureobasidium leucospermi*), D3 (*Aureobasidium melanogenum*), D4 (*Naganishia diffluens*), MA1 (*Saccharomyces cerevisiae*), MA2 (*Candida sake*), MA4 (*Lachancea fermentati*), MA7 (*Curvibasidium pallidicorallinum*), P8 (*Schizosaccharomyces pombe*), S1 (*Saccharomycodes ludwigii*), S5 (*Brettanomyces bruxellensis*), T1 (*Zygosaccharomyces bailii*), T17 (*Candida ethanolica*) and Z1 (*Rhodotorula nothofagi*). Plates were incubated 3 days at 30°C in triplicates and representative plates were photographed and reported.

**pH associated stress.** The ability of starter cultures to tolerate pH-associated stress is an important attribute for potential starter cultures. This follows that during alcoholic fermentation, a variety of organic acids are produced which lowers the pH of the fermentation broth, which is associated with hindering their productivity [17, 18]. This attribute will only be important if local brewers do not use pH- controlled brewing equipment and processes. In industrial processing, tolerance to low pH is not important because bioreactors can regulate pH by pumping in a base into the fermentation broth as per set fermentation pH. However, use of low pH-stress tolerant starter cultures, to cater for uncontrolled brewing is an advantage to increase productivity of starter cultures. Our results show that 6 out of 13 isolates tolerates a very low pH of 2 although this pH is not ideal for any alcoholic beverage (Fig. 5). In our previous work, we recorded an average of pH of 3.1 ± 0.31 [6] in agreement to a range from 2.87 to 3.16 reported by Mapitse *et al*. (2014). Our current results suggest that all the strains tolerated a pH of 3 except T1 (*Z. bailii*), and Z1 (*R. nothofagi*). This suggests that most of these isolates have potential to be used as starter cultures for uncontrolled brewing processes.

**Fig. 6:**
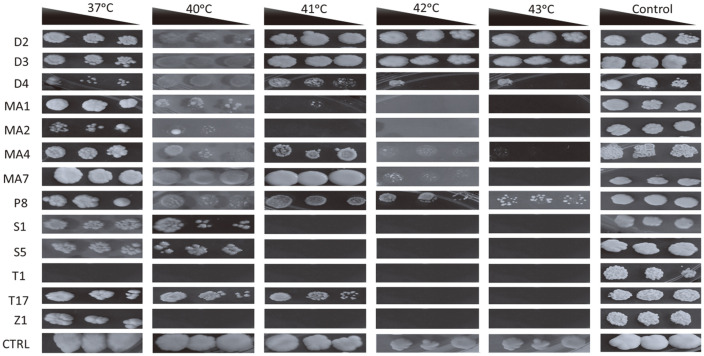
Thermo-tolerance of the isolates. The baker’s yeast (*S. cerevisiae*) was used as the control (CTRL) since it is a strain commonly found in *khadi*. The black triangle shows concentrations of the dilutions from left to right (OD_600nm_ 00.5, 0.25 and 0.125). Key: D2 (*Aureobasidium leucospermi*), D3 (*Aureobasidium melanogenum*), D4 (*Naganishia diffluens*), MA1 (*Saccharomyces cerevisiae*), MA2 (*Candida sake*), MA4 (*Lachancea fermentati*), MA7 (*Curvibasidium pallidicorallinum*), P8 (*Schizosaccharomyces pombe*), S1 (*Saccharomycodes ludwigii*), S5 (*Brettanomyces bruxellensis*), T1 (*Zygosaccharomyces bailii*), T17 (*Candida ethanolica*) and Z1 (*Rhodotorula nothofagi*). Plates were incubated 3 days at different temperatures in triplicates and representative plates were photographed and reported.

**Highly variable thermo-tolerance of *khadi* isolates.** Alcoholic fermentation is an exothermic process, which leads to an increase in temperatures in uncontrolled fermentation vessels. Just like pH, temperature tolerance could be ignored if fermentation is carried out in controlled fermentation vessels. However, use of temperature tolerant starter cultures is important to reduce the costs of cooling the fermentation vessels, mishap situations associated with thermal management faults and reduction in contamination [19]. Investigation of thermo-tolerance of potential starter cultures suggest all the strains, except for isolates T1 (*Z. bailii*), and Z1 (*R. nothofagi*)(Fig. 6), tolerated temperatures of 37°C and 40°C. Higher temperatures were inhibitory for most of the isolates except five (D2 [*A. leucospermi*], D3 [*A. melanogenum*], D4 [*N. diffluens*], MA4 [*L. fermentati*] and P8 [*S. pombe*]). The control yeast was also able to grow at temperature of 43°C. These results suggest that these isolates have potential to be used for *khadi* production at higher temperatures.

### Lachancea Fermentati as the Best Isolate for Brewing of *Khadi*

**Fig. 7:**
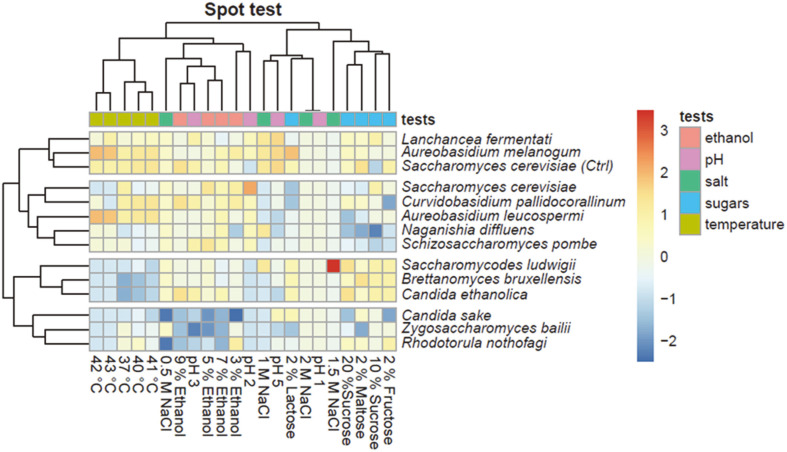
Heatmap presenting a summary of the stress tolerance experiment. All strains were grown in Yeast Peptone (YP) media supplemented with 2% of sole carbon source (Fructose, Maltose and Lactose) to evaluate the ability to utilize each of the carbon sources. Higher concentrations of sucrose (10 and 20%), different concentrations of ethanol (3, 5, 7 and 9%), different pH (2, 3, 4, and 5), different temperatures (37, 40, 42 and 43°C), different NaCl concentrations (5 and 10%) and higher concentrations of sucrose (10 and 20%) were also used to test for their tolerance to respective stresses.

*Lachancea fermentati* produced the highest amount of ethanol at an elevated fermentation rate 0.075 ml/h, grew well on media of pH 2, 1 M NaCl, 9% ethanol and at a temperature of 43°C (Fig. 7). In agreement with many studies, *L. fermentati* has been isolated from wine [20], cachaça [21], and water kefir [22] as well as non-fermented beverages such as coconut water and fruit juices [23]. This species is known to produce the fruity esters, which impact a characteristic fruity aroma to alcoholic brews [24-26] and associated with these specific aromas: higher alcohols, isoamyl alcohol, propanol and 2,3-butanodiol [25, 26]. We noted that in our work *L. fermentati* was isolated from Maun 2, Maun 3 and Palapye 3 *khadi* samples with distinct aroma profiles (Fig. 7) suggesting its contribution in the flavour/aroma of *khadi*.

It can also be observed be observed that *S. cerevisiae* (control strain) has a similar to the *L. fermentati* (Fig. 7). These 2 isolates are falling into the same cluster showing similar growth pattern. On the other hand, isolates *C. sake*, *Z. bailii* and *R. nothofagi* (fourth cluster) have an opposite growth behaviour to the first cluster. These 3 isolates averagely showed poor growth on the different stress tests.

### Fruity Aroma Characteristics Suggestive of Contribution of *L. fermentati* in 18 *khadi* Samples

**Fig. 8:**
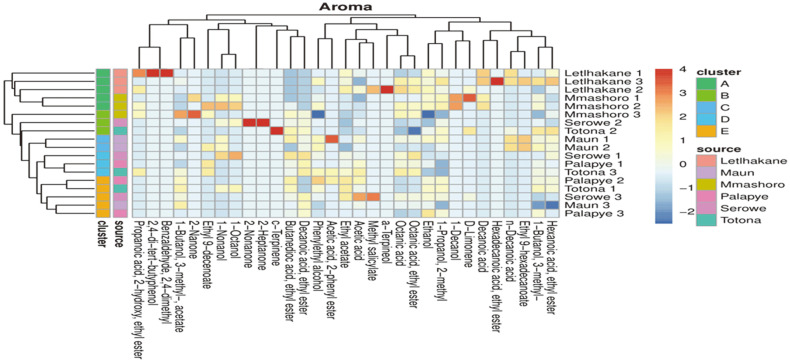
Distinct volatile organic compounds detected from *khadi* samples. The figure shows that the aromas could be clustered in 5 groups based on aroma similarities among the *khadi* samples. Cluster A are primarily esters and benzenic compounds, Cluster B are mainly esters, ketones and alcohols, Cluster C is mainly esters and alcohols, Cluster D is mainly esters and Cluster E is primarily esters and alcohols.

*Khadi* has a distinct unique flavour and taste characterised by fruity or floral aroma indicative of the presence of a variety of volatile organic compounds. We were prompted to perform an exhaustive study of the volatile compounds in *khadi* to track the aromatic signature of *L. fermentati* as a second step for selection of the best starter culture for commercialisation of *khadi*. Figure 8 shows volatile compounds identified among 18 *khadi* samples. Our results suggest that *khadi* samples from different brewers clustered into 5 groups (A - E). Our results suggest that the following volatile compounds were common in all the *khadi* samples; ethyl acetate, ethanol, octanoic acid, 1- butanol,3-methyl-,acetate, 1-butanol,3-methyl- and phenylethyl alcohol. Interestingly, all these 5 clusters had esters as the common and major aroma compounds, which probably accounts for the fruity aroma of *khadi*. This fruity and floral aromatic tones is in agreement with volatile compounds produced by yeasts in the *Lachancea* genus [24-26].

*L. fermentati* was isolated from Maun 2 (cluster E), Maun 3 (cluster E) and Palapye 3 (cluster D). The aroma profile of *khadi* from Maun 2 is made up of hexanoic acid-ethyl ester (sweet, fruity, pineapple, waxy, green banana), 1-butanol-3-methyl- (fusel, alcoholic, pungent, ethereal, cognac, fruity, banana and molasses), ethyl 9-hexadecanoate (fruity wine-like flavour), n-decanoic acid (unpleasant, rancid, sour, fatty, citrus), phenylethyl alcohol (sweet, floral, fresh and bready with a rosy honey nuance), 1-nonanol (oily, fatty, ethereal, slightly floral-fruity) and ethyl 9-decanoate (fruity apple flavour) (Fig. 8). The majority of the aromas from Maun 2 are esters which are generally known to be responsible for the fruity aroma of alcoholic beverages. The production of these esters could be due to *Lachancea fermentati* as research shows that *Lachancea* spp. are well-known producers of fruity esters, which impart a characteristic fruity flavor to alcoholic brews [24-26].

The aroma profile of Maun 3 *khadi* was made up of 1-butanol-3-methyl-acetate (sweet, fruity, estery, banana-and pear-like) and decanoic acid, ethyl ester (fruity apple flavour) (Fig. 8). The consortium of this beverage consisted of *L. fermentati* and *Curvibasidium pallidicorallinum* suggesting the contribution of these species in the overall fruity aroma of *khadi*. As for Palapye 3 aroma profile, the aromas were 1-butanol-3-methyl (fusel, alcoholic, pungent, ethereal, cognac, fruity, banana and molasses), 1-propanol-2-methyl (alcoholic, fermented, weak fusel, tequila like, musty, yeasty, with a slightly sweet fruity nuance of apple and pear), ethanol (strong, alcoholic, ethereal and medical) and decanoic acid, ethyl ester (fruity apple flavour) (Fig. 8). The yeast diversity of *khadi* sample from Palapye 3 was made up of *S. cerevisiae* and *L. fermentati*.

### Other Characteristic Aromas Suggestive of Contribution of Yeasts Other than *L. fermentati* Suggests a Mixed Culture Fermentation Could Be Considered as Starter Cultures

The aroma profiling results further show that *khadi* production can also utilize mixed culture fermentation. The other yeasts isolated from *khadi* could produce other aromas that contribute to the final distinct aroma of this alcoholic beverage. For instance, ethyl acetate (detected in all the samples) has been reported to be produced by *Brettanomyces bruxellensis* [27, 28], *Pichia kudriavzevii* [29], *S. cerevisiae* [30], *S. ludwigii* [31, 32] and *Lachancea fermentati* [26]. Such isolates have been found to be present in *khadi* samples such as *S. cerevisiae* (from both Palapye 2 and Tonota 1) and *S. ludwigii* (from Tonota 1). The other aroma compound of interest are organic acids which are responsible for the rapid acidification of alcoholic beverages [33] and the organic acid of detected in all the samples being octanoic acid (Fig. 8). The production of octanoic acid in beverages has been linked to the presence of *S. cerevisiae* [34], which was previously reported to be isolated in the majority of the *khadi* samples. Octanoic acids are responsible for the fresh notes in alcoholic beverages [35].

This study presents the potential of *L. fermentati* as starter culture for commercialization of the prominent traditional alcoholic beverage in Botswana. Although our results suggest there are distinct aromas of *khadi* from different producers countrywide, the characteristic fruity aroma is *khadi* could be due to this yeast. This work highlights that majority of the isolates presented in this work could also be used as mixed cultures with *L. fermentati*. Further studies to test for the functionality of the mixed consortia and in lab *khadi* chemical profiling could be useful for development of starter cultures for a consistent product quality towards commercialization of *khadi*.

## Supplemental Materials

Supplementary data for this paper are available on-line only at http://jmb.or.kr.

## Figures and Tables

**Table 1:** Yeasts isolates and controls used in this study.

Isolate code	Isolate identity	Source
D2	*Aureobasidium leucospermi*	*Grewia flava* fruits
D3	*Aureobasidium melanogenum*	*Grewia flava* fruits
D4	*Naganishia diffluens*	*Grewia flava* fruits
MA1	*Saccharomyces cerevisiae*	*Khadi*
MA2	*Candida sake*	*Khadi*
MA4	*Lachancea fermentati*	*Khadi*
MA7	*Curvibasidium pallidicorallinum*	*Khadi*
P8	*Schizosaccharomyces pombe*	*Khadi*
S1	*Saccharomycodes ludwigii*	*Khadi*
S5	*Brettanomyces bruxellensis*	*Khadi*
T1	*Zygosaccharomyces bailii*	*Khadi*
T17	*Candida ethanolica*	*Khadi*
Z1	*Rhodotorula nothofagi*	*Khadi*
Ale yeast	*Saccharomyces cerevisiae*	Commercial Ale yeast (SafAle T58, Fermentis, France)
Baker’s Yeast	*Saccharomyces cerevisiae*	Commercial Baker’s yeast (Anchor Yeast, South Africa)
Lager yeast	*Saccharomyces pastorianus*	Commercial Lager yeast (Lallemand Brewing, Austria)
Wine yeast	*Saccharomyces cerevisiae*	Commercial Wine yeast (Lalvin EC-1118, Lallemand Brewing, Austria)

Data extracted from Motlhanka *et al*, 2020.
