# Supplementary material for: Functional Characterization of khadi Yeasts Isolates for Selection of Starter Cultures
Source: J Microbiol Biotechnol. 2021 Dec 1;32(3):307–16. doi: 10.4014/jmb.2109.09003 (PMC9628858; doi:10.4014/jmb.2109.09003)
Supplement: Supplementary file 1 [file jmb-32-3-307-supple.pdf]

J. Microbiol. Biotechnol.
https://doi.org/10.4014/jmb.2109.09003

Supplementary Tables

Functional Characterization of *khadi* Yeasts Isolates for Selection of Starter Cultures

Koketso Motlhanka*, Kebaneilwe Lebani, Mar Garcia-Aloy and Nerve Zhou

Department of Biological Sciences and Biotechnology, Botswana International University of Science and Technology, Private Bag 16, Central District, Palapye, Botswana;

** Food Quality and Nutrition Department, Fondazione Edmund Mach, IASMA Research and Innovation Centre, via E. Mach 1, 38010 San Michele*

Table A1. Univariate tests of significance for CO_2 production rate

Effect	Univariate Tests of Significance for CO2 production rate (mL /hr) (CO2_Production_rate) Sigma-restricted parameterization Effective hypothesis decomposition				
	SS	Degr. of Freedom	MS	F	p
Intercept	0.103537	1	0.103537	16553.23	0.00
Treatment	0.012997	16	0.000812	129.87	0.00
Error	0.000319	51	0.000006		

Table A2. Post-hoc Turkey HSD data for CO2 production rate

Cell No.	Tukey HSD test; variable CO2 production rate (mL /hr) (CO2_Production_rate) Approximate Probabilities for Post Hoc Tests Error: Between MS = .00001, df = 51.000																	
	Treatment	{1} .06005	{2} .05157	{3} .04745	{4} .03897	{5} .07593	{6} .02742	{7} .03430	{8} .03882	{9} .02552	{10} .03277	{11} .02668	{12} .02645	{13} .01913	{14} .03680	{15} .04883	{16} .03920	{17} .03345
1	*Aureobasidium leucospermi*		0.001705	0.000173	0.000173	0.000173	0.000173	0.000173	0.000173	0.000173	0.000173	0.000173	0.000173	0.000173	0.000173	0.000177	0.000173	0.000173
2	*Naganishia diffluens*	0.001705		0.631170	0.000173	0.000173	0.000173	0.000173	0.000173	0.000173	0.000173	0.000173	0.000173	0.000173	0.000173	0.976025	0.000173	0.000173
3	*Saccharomyces cerevisiae*	0.000173	0.631170		0.001705	0.000173	0.000173	0.000173	0.001326	0.000173	0.000173	0.000173	0.000173	0.000173	0.000189	0.999993	0.002507	0.000173
4	*Candida sake*	0.000173	0.000173	0.001705		0.000173	0.000175	0.418657	1.000000	0.000173	0.070402	0.000173	0.000173	0.000173	0.997793	0.000268	1.000000	0.171687
5	*Lachancea fermentati*	0.000173	0.000173	0.000173	0.000173		0.000173	0.000173	0.000173	0.000173	0.000173	0.000173	0.000173	0.000173	0.000173	0.000173	0.000173	0.000173
6	*Curvibasidium pallidicorallinum*	0.000173	0.000173	0.000173	0.000175	0.000173		0.025463	0.000176	0.999550	0.211041	1.000000	1.000000	0.002300	0.000436	0.000173	0.000174	0.089874
7	*Schizosaccharomyces pombe*	0.000173	0.000173	0.000173	0.418657	0.000173	0.025463		0.474894	0.001039	0.999973	0.007383	0.005025	0.000173	0.990301	0.000173	0.340266	1.000000
8	*Saccharomycodes ludwigii*	0.000173	0.000173	0.001326	1.000000	0.000173	0.000176	0.474894		0.000173	0.086839	0.000174	0.000173	0.000173	0.999039	0.000242	1.000000	0.205048
9	*Brettanomyces bruxellensis*	0.000173	0.000173	0.000173	0.000173	0.000173	0.999550	0.001039	0.000173		0.013868	0.999999	1.000000	0.052657	0.000177	0.000173	0.000173	0.004409
10	*Zygosaccharomyces bailii*	0.000173	0.000173	0.000173	0.070402	0.000173	0.211041	0.999973	0.086839	0.013868		0.081027	0.058776	0.000173	0.669730	0.000173	0.050748	1.000000
11	*Candida ethanolica*	0.000173	0.000173	0.000173	0.000173	0.000173	1.000000	0.007383	0.000174	0.999999	0.081027		1.000000	0.008385	0.000226	0.000173	0.000173	0.029794
12	*Rhodotorula nothofagi*	0.000173	0.000173	0.000173	0.000173	0.000173	1.000000	0.005025	0.000173	1.000000	0.058776	1.000000		0.012250	0.000205	0.000173	0.000173	0.020866
13	*Aureobasidium melanogenum*	0.000173	0.000173	0.000173	0.000173	0.000173	0.002300	0.000173	0.000173	0.052657	0.000173	0.008385	0.012250		0.000173	0.000173	0.000173	0.000173

14	Baker's yeast	0.000173	0.000173	0.000189	0.997793	0.000173	0.000436	0.990301	0.999039	0.000177	0.669730	0.000226	0.000205	0.000173		0.000174	0.993599	0.884909
15	Ale yeast	0.000177	0.976025	0.999993	0.000268	0.000173	0.000173	0.000173	0.000242	0.000173	0.000173	0.000173	0.000173	0.000173	0.000174		0.000333	0.000173
16	Lager yeast	0.000173	0.000173	0.002507	1.000000	0.000173	0.000174	0.340266	1.000000	0.000173	0.050748	0.000173	0.000173	0.000173	0.993599	0.000333		0.129557
17	Wine yeast	0.000173	0.000173	0.000173	0.171687	0.000173	0.089874	1.000000	0.205048	0.004409	1.000000	0.029794	0.020866	0.000173	0.884909	0.000173	0.129557	

Table A3. Univariate tests of significance for total CO2 accumulated by different yeasts

Effect	Univariate Tests of Significance for CO2 accumulated (mL) (Total CO2 accumulated (mL)) Sigma-restricted parameterization Effective hypothesis decomposition				
	SS	Degr. of Freedom	MS	F	p
Intercept	55689.94	1	55689.94	59636.47	0.000000
Treatment	35.93	16	2.25	2.41	0.009079
Error	47.62	51	0.93		

Table A4. Post-hoc Turkey HSD data for total CO_2 accumulated by different yeasts

	Tukey HSD test; variable CO2 accumulated (mL) (Total CO2 accumulated (mL)) Approximate Probabilities for Post Hoc Tests Error: Between MS = .93382, df = 51.000																	
Cell No.	Treatment	{1} 26.375	{2} 29.500	{3} 28.375	{4} 29.125	{5} 28.625	{6} 29.250	{7} 29.000	{8} 28.875	{9} 29.000	{10} 28.375	{11} 28.625	{12} 27.875	{13} 27.750	{14} 29.125	{15} 28.875	{16} 28.500	{17} 29.250
1	*Aureobasidium leucospermi*		0.003331	0.256206	0.017262	0.117816	0.010102	0.028936	0.047453	0.028936	0.256206	0.117816	0.722950	0.829791	0.017262	0.047453	0.177019	0.010102
2	*Naganishia diffluens*	0.003331		0.960560	1.000000	0.996558	1.000000	0.999997	0.999941	0.999997	0.960560	0.996558	0.599628	0.473328	1.000000	0.999941	0.986395	1.000000
3	*Saccharomyces cerevisiae*	0.256206	0.960560		0.999418	1.000000	0.996558	0.999941	0.999997	0.999941	1.000000	1.000000	0.999997	0.999941	0.999418	0.999997	1.000000	0.996558
4	*Candida sake*	0.017262	1.000000	0.999418		0.999997	1.000000	1.000000	1.000000	1.000000	0.999418	0.999997	0.909973	0.829791	1.000000	1.000000	0.999941	1.000000
5	*Lachancea fermentati*	0.117816	0.996558	1.000000	0.999997		0.999941	1.000000	1.000000	1.000000	1.000000	1.000000	0.999418	0.996558	0.999997	1.000000	1.000000	0.999941
6	*Curvibasidium pallidicorallinum*	0.010102	1.000000	0.996558	1.000000	0.999941		1.000000	1.000000	1.000000	0.996558	0.999941	0.829791	0.722950	1.000000	1.000000	0.999418	1.000000
7	*Schizosaccharomyces pombe*	0.028936	0.999997	0.999941	1.000000	1.000000	1.000000		1.000000	1.000000	0.999941	1.000000	0.960560	0.909973	1.000000	1.000000	0.999997	1.000000
8	*Saccharomycodes ludwigii*	0.047453	0.999941	0.999997	1.000000	1.000000	1.000000	1.000000		1.000000	0.999997	1.000000	0.986395	0.960560	1.000000	1.000000	1.000000	1.000000
9	*Brettanomyces bruxellensis*	0.028936	0.999997	0.999941	1.000000	1.000000	1.000000	1.000000	1.000000		0.999941	1.000000	0.960560	0.909973	1.000000	1.000000	0.999997	1.000000
10	*Zygosaccharomyces bailii*	0.256206	0.960560	1.000000	0.999418	1.000000	0.996558	0.999941	0.999997	0.999941		1.000000	0.999997	0.999941	0.999418	0.999997	1.000000	0.996558
11	*Candida ethanolica*	0.117816	0.996558	1.000000	0.999997	1.000000	0.999941	1.000000	1.000000	1.000000	1.000000		0.999418	0.996558	0.999997	1.000000	1.000000	0.999941
12	*Rhodotorula nothofagi*	0.722950	0.599628	0.999997	0.909973	0.999418	0.829791	0.960560	0.986395	0.960560	0.999997	0.999418		1.000000	0.909973	0.986395	0.999941	0.829791
13	*Aureobasidium melanogenum*	0.829791	0.473328	0.999941	0.829791	0.996558	0.722950	0.909973	0.960560	0.909973	0.999941	0.996558	1.000000		0.829791	0.960560	0.999418	0.722950

14	Baker's yeast	0.017262	1.000000	0.999418	1.000000	0.999997	1.000000	1.000000	1.000000	1.000000	0.999418	0.999997	0.909973	0.829791		1.000000	0.999941	1.000000
15	Ale yeast	0.047453	0.999941	0.999997	1.000000	1.000000	1.000000	1.000000	1.000000	1.000000	0.999997	1.000000	0.986395	0.960560	1.000000		1.000000	1.000000
16	Lager yeast	0.177019	0.986395	1.000000	0.999941	1.000000	0.999418	0.999997	1.000000	0.999997	1.000000	1.000000	0.999941	0.999418	0.999941	1.000000		0.999418
17	Wine yeast	0.010102	1.000000	0.996558	1.000000	0.999941	1.000000	1.000000	1.000000	1.000000	0.996558	0.999941	0.829791	0.722950	1.000000	1.000000	0.999418	

Table A5: The stress tolerance of yeast isolates from *Grewia flava* fruits and *khadi*.

Samples	Sugars (%)					Ethanol (%)				Salt (M)				pH				Temperature (°C)				
	**YPS (10)**	**YPS (20)**	**YPL (2)**	**YPF (2)**	**YPM (2)**	**3**	**5**	**7**	**9**	**0.5**	**1.0**	**1.5**	**2.0**	**1**	**2**	**3**	**5**	**37**	**40**	**41**	**42**	**43**
**D2**	**++**	**-**	**+**	**++**	**+**	**++**	**++**	**++**	**+**	**+**	**-**	**-**	**-**	**-**	**+**	**++**	**-**	**+++**	**+++**	**+++**	**+++**	**+++**
**D3**	**++**	**++**	**+++**	**++**	**++**	**+++**	**++**	**++**	**+**	**+**	**+**	**-**	**-**	**-**	**+**	**++**	**+++**	**+++**	**+++**	**+++**	**+++**	**+++**
**D4**	**-**	**-**	**+**	**+**	**-**	**+**	**++**	**++**	**+**	**+**	**+**	**-**	**-**	**-**	**+**	**++**	**-**	**+**	**+**	**+**	**+**	**+**
**MA1**	**+++**	**+**	**-**	**++**	**+**	**+++**	**+++**	**++**	**+**	**+**	**-**	**-**	**-**	**-**	**++**	**++**	**+**	**+++**	**+**	**+**	**-**	**-**
**MA2**	**++**	**-**	**++**	**-**	**++**	**-**	**-**	**-**	**-**	**-**	**-**	**-**	**-**	**-**	**-**	**+**	**++**	**+**	**+**	**-**	**-**	**-**
**MA4**	**+++**	**++**	**+**	**++**	**++**	**++**	**++**	**+**	**+**	**+**	**+**	**-**	**-**	**-**	**+**	**+++**	**+++**	**++**	**++**	**++**	**+**	**+**
**MA7**	**++**	**++**	**-**	**-**	**++**	**+++**	**++**	**++**	**++**	**+**	**-**	**-**	**-**	**-**	**+**	**+++**	**+**	**+++**	**+++**	**+++**	**+**	**-**
**P8**	**+**	**+**	**+**	**+**	**+**	**++**	**+++**	**++**	**+**	**+**	**-**	**-**	**-**	**-**	**+**	**+++**	**+**	**++**	**+**	**+**	**+**	**+**

**S1**	+++	+++	++	+++	++	++	++	+	+	+	+	+	-	-	-	++	+	+	+	-	-	-
**S5**	+++	++	++	+++	+++	++	++	+	+	+	-	-	-	-	-	++	+	-	-	-	-	-
**T1**	++	+	-	++	-	++	-	-	-	+	-	-	-	-	-	-	-	++	+	+	-	-
**T17**	+++	+++	++	+++	++	++	++	++	++	+	-	-	-	-	-	+++	-	-	-	-	-	-
**Z1**	++	++	+	+++	++	+++	+	-	-	-	-	-	-	-	-	+	+	++	++	+	-	-
**Ctrl**	+	++	++	+++	+++	++	++	++	++	+	+	-	-	-	-	+++	+++	+++	+++	+++	++	++

Table A6: The averages of the fermentation profiling data

Time	*Aureobasidium leucospermi*	*Naganishia diffluens*	*Saccharomyces cerevisiae*	*Candida sake*	*Lachancea fermentati*	*Curvibasidium pallidicorallinum*	*Schizosaccharomyces pombe*	*Saccharomycodes ludwigii*	*Brettanomyces bruxellensis*	*Zygosaccharomyces bailii*	*Candida ethanolica*	*Rhodotorula nothofagi*	*Aureobasidium melanogenum*	Control
0	3.75	3.75	3.75	3.6	3.65	3.75	3.775	3.6	3.7	3.875	3.75	3.75	3.75	3.75
30	4	3.75	3.75	3.6	4.525	5.25	3.775	5.125	3.725	3.875	3.75	3.75	3.75	3.75
60	5.875	5.25	3.75	5.125	5.525	9.875	3.775	6	4.625	3.875	3.75	3.75	3.75	7.5
90	11.75	10.5	3.75	7.875	6.875	15.375	3.775	7.25	6.125	3.875	4.375	3.75	3.75	14
120	15.75	13.625	3.75	11.125	8.125	21.375	3.775	8.5	9	3.875	5	3.75	3.75	20
150	20	17.125	3.75	13.5	10	26.25	3.775	10.625	11.5	4	7	3.75	3.75	24.875
180	23.625	20.625	3.75	16.25	12.25	28.625	4.125	12.75	13.5	4.25	9.375	3.875	3.75	29.125
210	27	23.375	3.75	20	15.75		4.75	15.75	15.875	5.5	11.75	4.125	3.75	
240		26.875	3.75	24.75	19		5.375	18.625	18.5	6.625	14.5	4.875	4.5	
270		29.5	3.75	28.5	22.125		8.75	21.5	22.375	8.125	17.25	6.875	7.25	
30			3.75		26.37		12	23.625	25.75	10.625	19.25	9.875	11.5	

0				5									
330		6.625		29.125		16.25	25.375	28.875	12.375	21.625	13.75	16.625	
360		12.3				20.5	27.125		15	23.75	17.125	21.5	
390		18.5				25.125	29		17.5	26.125	19.75	25.875	
420		24.45				29.25			21.125	28.375	23.875	27.75	
450		27.75							25.125		28.625		
480									29				

Table B1. Identified compounds in the alcoholic liquid sample Letlhakane 1, Lab ID 16-09-20-12.

Ret. Time	Compound Name	Area %	Match factor	Probability
2.32	Ethyl acetate	5.09	905	94.13
2.74	Ethanol	41.04	933	94.36
4.62	1-Propanol, 2-methyl-	0.63	935	94.65
5.01	1-Butanol, 3-methyl-, acetate	1.29	945	84.54
6.18	1-Butanol, 3-methyl-,	10.38	925	67.66
6.58	Hexanoic acid, ethyl ester	2.43	940	88.14
8.09	Propanoic acid, 2-hydroxy, ethyl ester	1.75	925	41.27
9.31	Octanoic acid, ethyl ester	3.51	933	89.42
9.62	Acetic acid	1.30	954	82.16
11.84	Decanoic acid	1.61	928	80.55
13.90	Benzaldehyde, 2,4-dimethyl-	2.78	907	27.72
14.90	Phenylethyl alcohol	3.21	950	90.61
16.37	Octanoic acid	2.99	926	92.37
18.35	n-Decanoic acid	1.78	941	88.68
18.70	2,4-Di-tert-butylphenol	1.46	936	62.82

Table B2. Identified compounds in the alcoholic liquid sample Letlhakane 2, Lab ID 16-09-20-7.

Ret. Time	Compound Name	Area %	Match factor	Probability

2.32	Ethyl acetate	6.25	938	96.96
2.69-2.78	Ethanol	44.43	911	92.98
4.69	1-Propanol, 2-methyl-	0.73	936	95.92
4.93	1-Butanol, 3-methyl-, acetate	1.29	945	84.54
5.91	D-Limonene	2.49	935	36.47
6.17	1-Butanol, 3-methyl-,	8.54	915	66.40
6.50	Hexanoic acid, ethyl ester	1.83	948	90.20
8.07	Propanoic acid, 2-hydroxy, ethyl ester	1.93	933	61.04
9.28	Octanoic acid, ethyl ester	8.64	938	90.35
9.59	Acetic acid	1.84	949	82.40
11.83	Decanoic acid	1.72	933	81.67
12.53	à-Terpineol	1.13	928	55.13
13.49	Methyl salicylate	0.48	939	87.05
14.90	Phenylethyl alcohol	2.85	948	84.96
16.37	Octanoic acid	1.92	927	92.12

Table B3. Identified compounds in the alcoholic liquid sample Letlhakane 3, Lab ID 16-09-20-9. Two possible identities are given for peaks at 8.10 and 13.91 min as the NIST library gave close match factors.

Ret. Time	Compound Name	Area %	Match factor	Probability
2.32	Ethyl acetate	4.32	935	96.39
2.72	Ethanol	37.21	912	91.72
4.59	1-Propanol, 2-methyl-	1.35	932	95.95
5.03	1-Butanol, 3-methyl-, acetate	1.83	935	82.33
6.19	1-Butanol, 3-methyl-,	16.99	930	68.79

6.59	Hexanoic acid, ethyl ester	3.83	933	87.45
8.10	Ethyl (S)-(-)-Lactate **Cf** Propanoic acid, 2-hydroxy, ethyl ester	1.01	937/935	42.19/38.91
9.33	Octanoic acid, ethyl ester	7.54	940	90.17
11.85	Decanoic acid	1.64	935	83.01
13.91	Oxirane, 2-methyl-2-phenyl- **Cf** Isochroman	1.61	837/ 835	34.45/31.78
14.91	Phenylethyl alcohol	5.06	932	86.08
16.38	Octanoic acid	2.31	920	91.75
18.17	Hexadecenoic acid, ethyl ester	0.53	916	79.65
18.35	n-Decanoic acid	1.78	941	88.68
18.42	Ethyl 9-hexadecenoate	056	941	63.28

Table B4. Identified compounds in the alcoholic liquid sample Maun 1, Lab ID 16-09-20-14. Two possible identities are given for peaks at 8.06 and 13.90 min as the NIST library gave close match factors.

Ret. Time	Compound Name	Area %	Match factor	Probability
2.23	Ethyl acetate	3.03	938	95.51
2.29	Ethyl acetate	3.14	933	96.32
2.65	Ethanol	40.79	907	91.90
4.55	1-Propanol, 2-methyl-	0.93	935	94.06
4.96	1-Butanol, 3-methyl-, acetate	2.67	935	88.82
6.13	1-Butanol, 3-methyl-,	12.64	902	65.30
6.54	Hexanoic acid, ethyl ester	2.44	945	89.95

8.06	Propanoic acid, 2-hydroxy, ethyl ester Cf Ethyl (S)-(-)-Lactate	1.64	914/907	41.06/31.45
9.29	Octanoic acid, ethyl ester	5.69	940	89.35
9.59	Acetic acid	1.77	938	82.55
11.83	Decanoic acid, ethyl ester	0.62	926	84.42
12.05	1-Nonanol	0.80	892	32.63
12.29	Butanedioc acid, diethyl ester	1.58	922	83.88
13.90	Acetic acid, 2-phenylethyl ester	0.70	949	66.20
14.91	Phenylethyl alcohol	5.17	953	87.60
16.37	Octanoic acid	1.57	932	90.57
18.17	Hexadecenoic acid, ethyl ester	0.53	916	79.65
18.35	n-Decanoic acid	1.78	941	88.68
18.42	Ethyl 9-hexadecenoate	056	941	63.28

Table B5. Identified compounds in the alcoholic liquid sample Maun 2, Lab ID 16-09-20-16. Two possible identities are given each for peaks at 5.01, 5.09, 5.69, 8.17 and 13.90 min as the NIST library gave close match factors.

Ret. Time	Compound Name	Area %	Match factor	Probability
2.21	Ethyl acetate	1.17	931	97.35
2.60	Ethanol	20.95	877	92.24
2.74	Ethanol	30.53	907	92.99
3.62	Trichloromethane	2.44	926	88.72
4.68	1-Propanol, 2-methyl	0.58	900	92.86

4.89	1-Butanol,3-methyl-, acetate	0.69	724	69.75
5.01	p-Xylene/ o-Xylene	1.98	945/940	36.57/29.68
5.09	o-Xylene/ p-Xylene	1.08	948/938	39.19/27.67
5.69	p-Xylene/ o-Xylene	1.12	932/927	35.45/27.14
6.32	1-Butanol, 3-methyl-,	14.36	933	66.69
6.45	Hexanoic acid, ethyl ester	2.27	929	84.23
8.17	Propanoic acid, 2-hydroxy, ethyl ester **Cf** Ethyl (S)-(-)-Lactate	1.17	897/882	47.20/28.60
9.26	Octanoic acid, ethyl ester	4.04	939	90.72
9.65	Acetic acid	1.19	945	82.26
11.83	Decanoic acid, ethyl ester	0.62	926	84.42
12.05	1-Nonanol	0.80	892	32.63
12.44	Ethyl 9-decenoate	0.51	901	74.00
13.90	Oxirane, 2-methyl-2-phenyl- **Cf** Isochroman	1.02	825/822	39.09/34.54
14.91	Phenylethyl alcohol	5.32	951	87.96
16.37	Octanoic acid	0.98	922	92.59
18.17	Hexadecenoic acid, ethyl ester	0.53	916	79.65
18.35	n-Decanoic acid	1.78	941	88.68
18.42	Ethyl 9-hexadecenoate	056	941	63.28

Table B6. Identified compounds in the alcoholic liquid sample Maun 3, Lab ID 16-09-20-4. Two possible identities are given each for peaks at 6.96 and 7.02 min as the NIST library gave close match factors.

Ret.	Compound Name	Area	Match	Probability

Time		%	factor	
2.11	Ethyl acetate	3.40	887	95.67
2.30	Ethyl acetate	1.56	921	96.50
2.55	Ethanol	30.72	912	96.06
2.75	Ethanol	5.37	917	96.01
4.87	1-Butanol, 3-methyl-, acetate	3.57	919	80.34
4.93	1-Butanol, 3-methyl-, acetate	3.77	928	80.81
6.14	1-Butanol, 3-methyl-,	6.58	913	62.27
6.96	Propanoic acid, 2-methyl-, 3-methylbutyl ester **cf** Butanoic acid, 3-methylbutyl ester	7.33	918/911	45.13/34.57
7.02	Butanoic acid, 3-methylbutyl ester cf Propanoic acid, 2-methyl-, 3-methylbutyl ester	14.49	907/904	42.17/37.26
8.08	Propanoic acid, 2-hydroxy, ethyl ester	1.38	903	39.95
9.31	Octanoic acid, ethyl ester	5.06	938	89.75
9.61	Acetic acid	1.42	949	82.58
11.84	Decanoic acid, ethyl ester	1.02	930	83.53
12.30	Butanedioic acid, diethyl ester	0.57	938	86.61
14.92	Phenylethyl alcohol	2.23	950	87.62
16.38	Octanoic acid	0.77	920	92.86

Table B7. Identified compounds in the alcoholic liquid sample Mmashoro 1, Lab ID 16-09-20-2. Two possible identities are given each for peaks at 8.08 and 13.90 min as the NIST library gave close match factors.

Ret.	Compound Name	Area	Match	Probability

Time		%	factor	
2.34	Ethyl acetate	2.77	936	95.65
2.73	Ethanol	39.24	917	92.52
4.60	1-Propanol, 2-methyl-	1.06	946	96.31
4.98	1-Butanol, 3-methyl-, acetate	0.68	930	84.65
6.01	D-Limonene	10.11	943	52.89
6.16	1-Butanol, 3-methyl-,	12.57	901	62.34
6.56	Hexanoic acid, ethyl ester	1.27	944	88.20
8.08	Ethyl (S)-(-)-Lactate **Cf** Propanoic acid, 2-hydroxy, ethyl ester	0.96	936/934	42.71/39.40
8.71	2-Nanone	1.09	929	77.99
9.31	Octanoic acid, ethyl ester	4.69	940	90.54
10.70	Pentanoic acid, 2-hydroxy-4-methyl-, ethyl ester	0.76	921	82.56
10.83	1-Octanol	0.71	960	58.56
11.84	Decanoic acid	1.08	928	82.06
12.06	1-Nonanol	0.96	935	30.03
13.23	1-Decanol	0.99	951	26.06
13.90	Benzaldehyde **Cf** Oxirane, 2-methyl-2-phenyl-	1.18	866/ 864	23.03/21.24
14.91	Phenylethyl alcohol	3.64	950	87.81
16.38	Octanoic acid	0.87	914	92.69

Table B8. Identified compounds in the alcoholic liquid sample Mmashoro 2, Lab ID 16-09-20-10. Two possible identities are given for the peak at 13.90 min as the NIST library gave close match factors.

Ret. Time	Compound Name	Area %	Match factor	Probability
2.34	Ethyl acetate	2.91	953	96.43
2.74	Ethanol	42.61	903	91.63
4.64	1-Propanol, 2-methyl-	1.16	933	96.02
4.96	1-Butanol, 3-methyl-, acetate	0.68	941	8392
6.16	1-Butanol, 3-methyl-,	13.13	910	60.91
6.53	Hexanoic acid, ethyl ester	1.50	936	89.26
8.07	Propanoic acid, 2-hydroxy, ethyl ester	1.21	907	39.55
8.16	1-Hexanol	0.75	904	66.87
9.29	Octanoic acid, ethyl ester	6.85	936	89.36
9.61	Acetic acid	0.74	937	84.30
10.59	Nonanoic acid, ethyl ester	0.59	898	80.79
10.69	Pentanoic acid, 2-hydroxy-4-methyl-, ethyl ester	0.85	908	78.41
10.83	1-Octanol	1.25	966	83.16
11.84	Decanoic acid	1.35	935	35.63
12.06	1-Nonanol	1.70	946	84.84
12.29	Butanedioic acid, diethyl ester	0.68	930	76.70
12.45	Ethyl 9-decenoate	0.79	909	31.88
13.23	1-Decanol	0.99	946	37.59
13.90	Oxirane, 2-methyl-2-phenyl- Cf Benzaldehyde, 2,4- dimethyl-	1.37	872/850	37.59/14.86
14.91	Phenylethyl alcohol	4.78	952	89.07
16.38	Octanoic acid	1.55	922	93.13

Table B9. Identified compounds in the alcoholic liquid sample Mmashoro 3, Lab ID 16-09-20-8. Two possible identities are given for the peak at 6.01 min as the NIST library gave close match factors.

Ret. Time	Compound Name	Area %	Match factor	Probability
2.11	Ethyl acetate	1.79	863	96.17
2.29	Ethyl acetate	0.77	923	96.00
2.54	Ethanol	1.16	926	94.62
2.70	Ethanol	0.68	923	96.00
4.91	1-Butanol, 3-ethyl-, acetate	5.31	921	79.76
4.97	1-Butanol, 3-ethyl-, acetate	6.66	918	79.76
6.01	D-Limonene Cf Limonene	2.03	915/905	22.81/16.11
6.11	1-Butanol, 3-methyl-,	7.87	904	60.46
6.54	Hexanoic acid, ethyl ester	1.87	941	89.97
7.01	Propanoic acid, 2-hydroxy, 3-methylbutyl ester	21.36	913	65.19
8.07	Propanoic acid, 2-hydroxy, ethyl ester	1.37	916	89.91
9.31	Octanoic acid, ethyl ester	7.05	941	90.79
9.62	Acetic acid	0.82	933	83.61
11.84	Decanoic acid, ethyl ester	0.75	932	83.67
12.30	Butanedioic acid, diethyl ester	1.20	928	84.28
12.46	Ethyl 9-decenoate	0.37	905	76.45
12.54	a-Terpineol	1.77	935	54.54

Table B10. Identified compounds in the alcoholic liquid sample Palapye 1, Lab ID 16-09-20-13. Two possible identities are given for the peak at 8.06 min as the NIST library gave close match factors.

Ret. Time	Compound Name	Area %	Match factor	Probability
2.11	Ethyl acetate	3.70	862	95.06
2.30	Ethyl acetate	3.44	932	96.29
2.53	Ethanol	29.96	927	93.90
2.71	Ethanol	13.78	934	96.94
4.90	1-Butanol, 3-methyl-, acetate	0.67	938	85.52
4.96	1-Butanol, 3-methyl-, acetate	1.53	935	83.81
6.11	1-Butanol, 3-methyl-,	11.83	917	69.62
6.53	Hexanoic acid, ethyl ester	1.68	947	89.12
8.06	Propanoic acid, 2-hydroxy, ethyl ester Cf Ethyl-(s)-(-)-lactate	2.02	934/919	46.35/28.00
9.30	Octanoic acid, ethyl ester	5.92	929	88.58
9.61	Acetic acid	1.91	944	82.24
10.69	Pentanoic acid, 2-hydroxy-4-methyl-, ethyl ester	0.70	918	81.62
10.83	1-Octanol	0.70	947	59.01
11.83	Decanoic acid, ethyl ester	1..20	932	85.29
12.29	Butanedioic acid, diethyl ester	0.84	920	84.41
12.45	Ethyl 9-decenoate	0.58	903	73.87
14.91	Phenylethyl alcohol	3.29	957	88.83
16.38	Octanoic acid	2.14	921	91.86
18.17	Hexadecanoic acid, ethyl ester	0.13	904	69.25

Table B11. Identified compounds in the alcoholic liquid sample Palapye 2, Lab ID 16-09-20-11. Two possible identities are given for the peak at 7.99 min as the NIST library gave close match factors.

Ret. Time	Compound Name	Area %	Match factor	Probability
2.23	Ethyl acetate	6.76	936	96.22
2.61	Ethanol	37.66	919	93.41
3.34	Acetonitrile	2.50	982	50.94
4.41	1-Propanol, 2-methyl	0.90	926	94.06
4.79	1-Butanol, 3-methyl-, acetate	2.80	940	88.22
6.01	1-Butanol, 3-methyl-	12.54	926	68.50
6.41	Hexanoic acid, ethyl ester	2.23	946	90.60
7.99	Propanoic acid, 2-hydroxy, ethyl ester Cf Ethyl-(s)-(-)-lactate	1.97	935/930	43.40/34.98
9.25	Octanoic acid, ethyl ester	3.89	929	88.34
9.57	Acetic acid	2.34	943	81.23
10.67	Pentanoic acid, 2-hydroxy-4-methyl-, ethyl ester	0.64	914	79.73
10.81	1-Octanol	0.59	947	55.65
12.05	1-Octanol	0.69	905	32.49
12.28	Butanedioic acid, diethyl ester	1.59	926	83.70
13.89	Acetic acid, 2-phenylethyl ester	0.68	915	59.33
14.20	Hexanoic acid	0.57	950	89.55
14.90	Phenylethyl alcohol	6.98	956	87.91
16.37	Octanoic acid	1.86	922	93.71

18.17	Hexadecanoic acid, ethyl ester	1.31	929	82.91

Table B12. Identified compounds in the alcoholic liquid sample Palapye 3, Lab ID 16-09-20-17. Two possible identities are given for the peak at 13.91 min as the NIST library gave close match factors.

Ret. Time	Compound Name	Area %	Match factor	Probability
1.95	Ethyl acetate	3.16	925	96.19
2.05	Ethyl acetate	4.11	936	97.17
2.40	Ethanol	53.38	919	93.72
4.38	1-Propanol, 2-methyl	0.83	931	94.54
4.85	1-Butanol, 3-methyl-, acetate	1.29	931	79.47
6.05	1-Butanol, 3-methyl-	13.09	917	67.36
6.47	Hexanoic acid, ethyl ester	1.61	928	84.67
8.04	Ethyl-(S)-(-)-lactate	1.45	936	42.29
9.29	Octanoic acid, ethyl ester	3.48	937	89.28
9.61	Acetic acid	1.04	940	84.24
10.69	Pentanoic acid, 2-hydroxy-4-methyl-, ethyl ester	0.62	917	84.72
11. 84	Decanoic acid, ethyl ester	0.86	934	83.02
12.29	Butanedioc acid, diethyl ester	0.72	915	85.02
13.91	Oxirane, 2-methyl-2-phenyl- Cf Benzaldehyde, 2,5- dimethyl-	0.92	854/846	23.006/
14.91	Phenylethyl alcohol	2.82	942	86.74
16.38	Octanoic acid	1.14	927	92.37

Table B13. Identified compounds in the alcoholic liquid sample Serowe 1, Lab ID 16-09-20-1. Two possible identities are given for the peak at 3.19 min as the NIST library gave close match factors.

Ret. Time	Compound Name	Area %	Match factor	Probability
1.95	Ethyl acetate	4.09	895	95.07
2.47	Ethanol	24.39	909	93.15
2.57	Ethanol	20.66	899	92.80
3.19	Methyl isocyanide Cf Acetonitrile	1.21	818/807	57.03/39.12
4.40	1-Propanol, 2-methyl	0.42	892	94.80
4.64	1-Butanol, 3-methyl-, acetate	1.50	925	79.98
6.02	1-Butanol, 3-methyl-	10.71	924	67.73
6.28	Hexanoic acid, ethyl ester	2.30	947	89.70
6.50	Butanoic acid, 3-methyl-,butyl ester	0.62	882	61.24
8.02	Ethyl-(S)-(-)-lactate	1.90	938	40.34
9.24	Octanoic acid, ethyl ester	6.09	941	90.51
9.60	Acetic acid	1.27	931	82.16
10.57	Nonanoic acid, ethyl ester	0.52	874	81.16
10.69	Pentanoic acid, 2-hydroxy-4-methyl-, ethyl ester	1.06	917	82.75
10.82	1-Octanol	0.73	943	59.58
11.83	Decanoic acid, ethyl ester	1.53	927	82.62
12.06	1-Nonanol	1.49	947	37.31
12.30	Butanedioic acid, diethyl ester	1.80	938	85.66
14.91	Phenylethyl alcohol	3.96	953	86.25
16.38	Octanoic acid	1.08	923	91.31

Table B14. Identified compounds in the alcoholic liquid sample Serowe 2, Lab ID 16-09-20-6.

Ret. Time	Compound Name	Area %	Match factor	Probability
2.24	Ethyl acetate	1.32	917	96.72
2.34	Ethyl acetate	6.59	943	96.64
2.66	Ethanol	15.21	931	96.18
2.74	Ethanol	33.80	915	92.64
4.56	1-Propanol, 2-methyl	0.69	932	94.74
5.00	1-Butanol, 3-methyl-, acetate	1.50	938	82.63
5.84	2-Heptanone	0.76	899	77.54
6.16	1-Butanol, 3-methyl-	11.18	929	71.02
6.56	Hexanoic acid, ethyl ester	1.29	950	89.88
8.08	Propanoic aid, 2-methyl 2-hydroxy-; ethyl ester	1.98	933	48.72
8.71	2-Nonanone	0.79	919	78.51
9.23	Benzene, 1,3-bis(1,1-dimethylethyl)-	0.52	943	84.80
9.31	Octanoic acid, ethyl ester	3.69	927	89.39
9.62	Acetic acid	1.17	947	82.83
10.70	Pentanoic acid, 2-hydroxy-4-methyl-, ethyl ester	0.86	897	76.11
11.84	Decanoic acid, ethyl ester	0.77	935	84.09
12.06	1-Nonanol	0.60	938	34.55
12.29	Butanedioic acid, diethyl ester	0.81	926	82.82
14.91	Phenylethyl alcohol	3.38	951	87.17
16.38	Octanoic acid	1.22	922	90.42

Table B15. Identified compounds in the alcoholic liquid sample Serowe 3, Lab ID 16-09-20-5. Two possible identities are given for the peak at 10.94 min as the NIST library gave close match factors.

Ret. Time	Compound Name	Area %	Match factor	Probability
2.33	Ethyl acetate	6.59	934	96.02
2.73	Ethanol	38.47	917	92.41
3.50	Acetonitrile	1.88	989	57.43
4.68	1-Propanol, 2-methyl	0.77	929	94.15
4.93	1-Butanol, 3-methyl-, acetate	2.21	927	81.74
5.91	D-Limonene	1.54	940	44.48
6.17	1-Butanol, 3-methyl-	8.97	927	65.31
6.50	Hexanoic acid, ethyl ester	1.43	933	84.43
8.08	Propanoic aid, 2-methyl 2-hydroxy-; ethyl ester	1.66	905	40.90
9.28	Octanoic acid, ethyl ester	5.74	940	90.10
9.58	Acetic acid	3.84	949	81.75
10.71	Linanool	0.61	889	65.63
10.94	Cyclohexanol,2-(1,1-dimethylethyl)- Cf 4-tert-Butylcyclohexyl acetate	1.40	857/855	33.95/31.32
11.83	Decanoic acid, ethyl ester	1.15	933	83.25
12.29	Butanedioic acid, diethyl ester	0.82	915	83.48
13.50	Methyl salicylate	0.61	943	85.41
14.91	Phenylethyl alcohol	3.55	955	86.49
16.38	Octanoic acid	1.61	924	93.40

Table B16. Identified compounds in the alcoholic liquid sample identified as Tonota 1, ID 16-09-2018. Two possible identities are given for the peak at 8.05 min as the NIST library gave close match factors.

Ret. Time	Compound Name	Area %	Match factor	Probability
2.28	Ethyl acetate	6.66	934	95.93
2.67	Ethanol	40.88	917	92.50
3.46	Acetonitrile	3.30	989	55.68
4.54	1-Propanol, 2-methyl	0..97	932	94.88
4.95	1-Butanol, 3-methyl-, acetate	3.43	937	84.80
6.13	1-Butanol, 3-methyl-	14.29	903	65.44
6.53	Hexanoic acid, ethyl ester	2.36	944	89.40
8.05	Ethyl(S)-(-)-lactate **Cf** Propanoic aid, 2-methyl 2-hydroxy-; ethyl ester	1.75	936/933	41.60/36.76
9.28	Octanoic acid, ethyl ester	3.89	939	90.86
9.59	Acetic acid	1.42	932	82.53
10.68	Pentanoic acid, 2-hydroxy-4-methyl-,ethyl ester	0.71	919	81.06
10.82	1-Octanol	0.62	960	63.54
12.06	1-Nonanol	0.72	906	31.39
12.29	Butanedioic acid, diethyl ester	1.32	927	83.27
13.90	Acetic acid, 2-phenylethyl ester	0.69	928	63.04
14.21	Hexanoic acid	0.52	935	83.74
14.91	Phenylethyl alcohol	4.55	950	86.80
16.38	Octanoic acid	1.59	920	1.96

Table B17. Identified compounds in the alcoholic liquid sample identified as Tonota 2, Lab ID 16-09-20-15. Two possible identities are given for the peak at 7.02 min as the NIST library gave close match factors.

Ret. Time	Compound Name	Area %	Match factor	Probability
2.04	Ethyl acetate	1.97	804	95.37
2.28	Ethyl acetate	1.50	934	96.52
2.48	Ethanol	31.63	912	94.35
2.66	Ethanol	8.54	921	92.25
4.46	1-Propanol, 2-methyl	0.93	932	95.77
4.82	1-Butanol, 3-methyl-, acetate	0.53	931	84.59
4.92	1-Butanol, 3-methyl-, acetate	1.01	932	78.85
5.95	D-Limonene	5.26	926	41.04
6.06	1-Butanol, 3-methyl	16.28	907	66.47
6.50	Hexanoic acid, ethyl ester	3.46	931	83.89
6.64	ç-Terpinene	0.61	917	34.96
7.02	p-cymene Cf o-Cymene	1.27	932/930	21.13/19.49
8.05	Propanoic acid, ethyl ester	1.41	927	39.93
9.29	Octanoic acid, 2-hydroxy-, ethyl ester	3.03	933	89.51
9.62	Acetic acid	0.73	927	89.56
10.71	Linanool	0.53	877	61.53
11.84	Decanoic acid, ethyl ester	0.90	938	83.63
12.30	Butanedioic acid, diethyl ester	1.48	936	85.65
13.87	Benzoic acid, 2-hydroxy-, ethyl ester	1.40	941	91.76
14.21	Hexanoic acid	0.53	943	89.90

14.91	Phenylethyl alcohol	3.10	952	87.63
16.39	Octanoic acid	1.64	923	93.11
18.36	n-Decanoic acid	0.51	928	86.99

Table B18. Identified compounds in the alcoholic liquid sample identified as Tonota 3, Lab ID 16-09-20-3.

Ret. Time	Compound Name	Area %	Match factor	Probability
2.29	Ethyl acetate	4.88	941	96.12
2.66	Ethanol	16.36	929	95.16
2.72	Ethanol	28.26	917	92.84
4.92	1-Butanol, 3-methyl-, acetate	2.40	921	86.83
6.23	1-Butanol, 3-methyl-	12.38	924	64.89
6.51	Hexanoic acid, ethyl ester	1.93	940	88.76
8.11	Propanoic aid, 2-hydroxy-; ethyl ester	1.87	926	40.84
9.30	Octanoic acid, ethyl ester	7.94	934	89.87
9.61	Acetic acid	2.59	948	82.37
11.83	Decanoic acid, ethyl ester	1.33	937	84.03
12.30	Butanedioic acid, diethyl ester	0.74	924	83.99
12.45	Ethyl 9-decenoate	0.54	905	75.05
13.90	Acetic acid, 2-phenylethyl ester	0.59	890	54.15
14.91	Phenylethyl alcohol	5.26	956	88.60
16.38	Octanoic acid	1.35	918	92.43
